# *In vitro* treatment of HepG2 cells with saturated fatty acids reproduces mitochondrial dysfunction found in nonalcoholic steatohepatitis

**DOI:** 10.1242/dmm.018234

**Published:** 2014-12-24

**Authors:** Inmaculada García-Ruiz, Pablo Solís-Muñoz, Daniel Fernández-Moreira, Teresa Muñoz-Yagüe, José A. Solís-Herruzo

**Affiliations:** 1 Research Center, Laboratory of Gastroenterology and Hepatology, University Hospital “12 de Octubre”, Complutense University, 28041-Madrid, Spain.; 2 Anglo-American Medical Unit, 28001-Madrid, Spain.; 3 Department of Bromatology and Food Hygiene, Military Center of Veterinary of Defense, 28024-Madrid, Spain.

**Keywords:** Mitochondrial respiratory chain, Nonalcoholic steatohepatitis, NADPH oxidase, Oxidative phosphorylation, Proteomic, Nitro-oxidative stress, OXPHOS

## Abstract

Activity of the oxidative phosphorylation system (OXPHOS) is decreased in humans and mice with nonalcoholic steatohepatitis. Nitro-oxidative stress seems to be involved in its pathogenesis. The aim of this study was to determine whether fatty acids are implicated in the pathogenesis of this mitochondrial defect. In HepG2 cells, we analyzed the effect of saturated (palmitic and stearic acids) and monounsaturated (oleic acid) fatty acids on: OXPHOS activity; levels of protein expression of OXPHOS complexes and their subunits; gene expression and half-life of OXPHOS complexes; nitro-oxidative stress; and NADPH oxidase gene expression and activity. We also studied the effects of inhibiting or silencing NADPH oxidase on the palmitic-acid-induced nitro-oxidative stress and subsequent OXPHOS inhibition. Exposure of cultured HepG2 cells to saturated fatty acids resulted in a significant decrease in the OXPHOS activity. This effect was prevented in the presence of a mimic of manganese superoxide dismutase. Palmitic acid reduced the amount of both fully-assembled OXPHOS complexes and of complex subunits. This reduction was due mainly to an accelerated degradation of these subunits, which was associated with a 3-tyrosine nitration of mitochondrial proteins. Pretreatment of cells with uric acid, an antiperoxynitrite agent, prevented protein degradation induced by palmitic acid. A reduced gene expression also contributed to decrease mitochondrial DNA (mtDNA)-encoded subunits. Saturated fatty acids induced oxidative stress and caused mtDNA oxidative damage. This effect was prevented by inhibiting NADPH oxidase. These acids activated NADPH oxidase gene expression and increased NADPH oxidase activity. Silencing this oxidase abrogated totally the inhibitory effect of palmitic acid on OXPHOS complex activity. We conclude that saturated fatty acids caused nitro-oxidative stress, reduced OXPHOS complex half-life and activity, and decreased gene expression of mtDNA-encoded subunits. These effects were mediated by activation of NADPH oxidase. That is, these acids reproduced mitochondrial dysfunction found in humans and animals with nonalcoholic steatohepatitis.

## INTRODUCTION

Nonalcoholic fatty liver disease (NAFLD) represents a spectrum of liver diseases extending from pure fatty liver through nonalcoholic steatohepatitis (NASH) to cirrhosis and hepatocarcinoma that occurs in individuals who do not consume a significant amount of alcohol (Matteoni et al., 1999). Although the pathogenesis of NAFLD remains undefined, the so-called ‘two hits’ model of pathogenesis has been proposed (Day and James, 1998). Whereas the ‘first hit’ involves the accumulation of fat in the liver, the ‘second hit’ includes oxidative stress resulting in inflammation, stellate cell activation, fibrogenesis and progression of NAFLD to NASH (Chitturi and Farrell, 2001). Mitochondrial dysfunction might play a crucial role in the induction of both ‘hits’, because mitochondria are involved in the β-oxidation of free fatty acids, and are the most important source of reactive oxygen species (ROS) (Fromenty et al., 2004). In previous studies, we have shown that oxidative phosphorylation (OXPHOS) is defective in individuals with NASH (Pérez-Carreras et al., 2003), in *ob/ob* mice with NAFLD (García-Ruiz et al., 2006) and in mice on a high-fat diet (García-Ruiz et al., 2014). We also demonstrated that this mitochondrial dysfunction can be prevented by treating mice with antioxidants and antiperoxinitrites, such as melatonin or uric acid, indicating that oxidative and nitrosative stress might play a crucial role in the pathogenesis of this defect. However, the cause of this stress remains unclear. Potential sources of nitro-oxidative stress are multiple, including cytochrome P450-2E1 (CYP2E1) (Weltman et al., 1998), nicotinamide adenine dinucleotide phosphate-oxidase or NADPH (nicotinamide adenine dinucleotide phosphate) oxidase (NADPHox) (De Minicis et al., 2006), mitochondrial electron transport chain (Fridovich, 2004) and xanthine oxidase (XDH) (Spiekermann et al., 2003). CYP2E1, a member of the oxido-reductase cytochrome family, can oxidize a variety of small molecules, including fatty acids (Caro and Cederbaum, 2004), to produce superoxide anions, a very potent reactive oxygen species (ROS). Activity and expression of this enzyme is increased in the liver of humans and animals with NAFLD (Weltman et al., 1998), and this increase correlates with the severity of NAFLD. NADPHox is a multiprotein complex found in all types of liver cells, including hepatocytes, that reduces molecular oxygen to superoxide and hydrogen peroxide (De Minicis et al., 2006). In a previous study, we have shown that mice with diet-induced NASH have elevated NADPHox gene expression and activity (García-Ruiz et al., 2014), and other authors have found the same changes in mice fed a methionine-choline-deficient diet (Greene et al., 2014). A number of factors can induce NADPHox activity, including free fatty acids (Hatanaka et al., 2013) and TNFα (Mohammed et al., 2013), among others. Considering that fatty acids are increased in the liver of obese mice (García-Ruiz et al., 2014), it might be possible that these acids are responsible for the increased NADPHox activity, the oxidative stress and eventually for the OXPHOS dysfunction found in individuals with NASH and in obese mice. OXPHOS dysfunction, in turn, might create a vicious cycle that would contribute to increase the oxidative stress.

**TRANSLATIONAL IMPACT**

**Clinical issue**

Nonalcoholic fatty liver disease (NAFLD) is a worldwide problem and its histopathological hallmarks represent the most frequent histological finding in individuals with abnormal liver tests in the Western countries. Although NAFLD pathogenesis remains poorly understood, previous works found that the disease is associated with decreased oxidative phosphorylation (OXPHOS), a mitochondrial metabolic pathway through which energy released by oxidation of nutrients is converted into ATP to supply energy to cell metabolism. Because mitochondria are involved in the oxidation of fatty acids and are important sources of reactive oxygen species (ROS), defective OXPHOS might contribute to the accumulation of fat in the liver and cause oxidative stress leading to steatohepatitis and cirrhosis. Previous works demonstrated a marked decrease in OXPHOS activity in mice fed a high-fat diet that seemed to be related with the nitro-oxidative stress. The present study aims to determine whether fatty acids are implicated in the pathogenesis of this mitochondrial defect and to elucidate the role played by the NADPH oxidase in the generation of oxidative stress and mitochondrial dysfunction.

**Results**

In this study, the authors used a human liver carcinoma cell line (HepG2) and found that, in these cells, saturated fatty acids cause a decrease in the OXPHOS activity that is due to a decrease in the amount of OXPHOS-complex subunits. These fatty acids provoke nitro-oxidative stress, cause 3-tyrosine nitration of mitochondrial proteins and accelerate the degradation of such proteins. Pretreatment of cells with an antiperoxynitrite agent prevented these effects. Oxidative damage of mitochondrial DNA also reduced the synthesis of mitochondrial OXPHOS subunits. NADPH oxidase seems to play a crucial role in the pathogenesis of the nitro-oxidative stress and OXPHOS dysfunction, because the effects of saturated fatty acids are prevented in the absence of NADPH oxidase activity. Moreover, these fatty acids activate NADPH oxidase gene expression and increase NADPH oxidase activity.

**Implications and future directions**

These results show that saturated fatty acids decrease OXPHOS enzymatic activity, owing to a decreased amount of OXPHOS complex subunits. These effects are mediated by fatty-acid-induced nitro-oxidative stress and by NADPH oxidase. The use of antioxidants or antiperoxynitrites can prevent these changes. Therefore, treatment with these agents or with inhibitors of the NADPH oxidase, as well as strategies for reducing hepatic free fatty acid concentration. might be useful in preventing the progression of NAFLD in humans.

The aims of this study were to determine whether fatty acids are implicated in the pathogenesis of this mitochondrial defect and to know the role played by NADPHox in the generation of this dysfunction.

## RESULTS

### Saturated fatty acids decreased OXPHOS enzyme activity

**Fig. 1.**
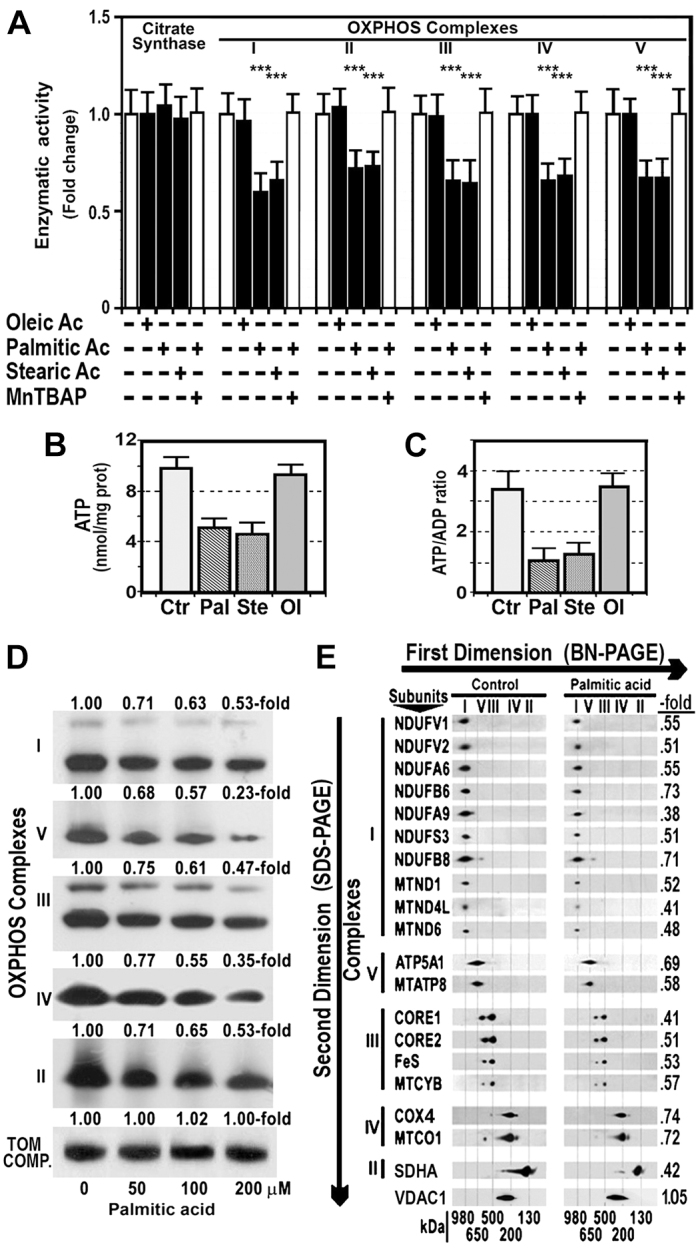
**Saturated fatty acids decreased enzyme activity of the OXPHOS complexes, ATP content, and the amount of fully-assembled complexes and their subunits.** (A) Enzyme activity of the OXPHOS complexes and citrate synthase was measured in HepG2 cells treated for 24 hours with 200 μM oleic acid, 200 μM palmitic acid, 200 μM stearic acid or 4 mM MnTBAP, normalized to the level of the specific activity of citrate synthase, and expressed as fold of the activity in untreated cells. Values are expressed as mean ± s.d. ***P<0.001 compared with untreated cells. (B) ATP content in control HepG2 cells (Ctr) and cells exposed 200 μM palmitic acid (Pal), 200 μM stearic acid (Ste) or 200 μM oleic acid (Ol) for 24 hours. (C) ATP:ADP ratio in HepG2 cells treated with 200 μM palmitic acid (Pal), 200 μM stearic acid (Ste) or 200 μM oleic acid (Ol) for 24 hours. (D) Mitochondrial complexes isolated from HepG2 cells and treated with increasing doses of palmitic acid were separated on a BN-PAGE system. Western blot analysis of mitochondrial proteins was performed using antibody against complex I subunit NDUFA9, complex II subunit SDHA, complex III subunit core 2 protein, complex IV subunit MTCO1, complex V subunit ATP5A1, and TOM complex subunit TOM20. Expression of TOM complex (TOM COMP.) was used as loading control. (E) Mitochondrial complexes extracted from HepG2 cells treated with 200 μM palmitic acid for 24 hours were separated in the first dimension using BN-PAGE and in the second dimension using SDS-PAGE. Presence of individual subunits of these complexes was identified by immunoblotting using appropriate antibodies. Expression of VDAC1 was used as a loading control. ‘-fold’ indicates the amount of subunit in palmitic-acid-treated cells divided by the amount of the same subunit in control cells.

Treatment of HepG2 cells with 200 μM oleic acid, a monounsaturated fatty acid, did not significantly alter activity of OXPHOS complexes. However, treatment of these cells with the same doses of palmitic or stearic acids, two saturated fatty acids, decreased enzyme activity of these complexes to about 66.8±4.3% of control activity (Fig. 1A). These effects were abrogated by pretreating cells with 4 mM manganese [III] tetrakis (5,10,15,20 benzoic acid) porphyrin (MnTBAP), a mimic of manganese superoxide dismutase. Because the final product of OXPHOS is ATP, we measured the ATP content in HepG2 cells treated with 200 μM palmitic, stearic or oleic acid for 24 hours. As Fig. 1B shows, palmitic and stearic acids decreased cellular ATP from 9.86±0.33 nmol/mg protein to 5.16±0.3 or 4.67±0.4 nmol/mg protein (*P*<0.01), respectively. Treatment of cells with 200 μM oleic acid did not affect cellular ATP. Likewise, the ATP:ADP ratio was also significantly decreased in cells treated with palmitic or stearic acids, but not in those treated with oleic acid (Fig. 1C).

### Palmitic acid decreased fully-assembled OXPHOS complexes and complex subunits

The first-dimension BN-PAGE system illustrates that fully-assembled OXPHOS complexes decreased in a dose-dependent manner in HepG2 cells treated with increasing doses of palmitic acid for 24 hours (Fig. 1D). To study how mitochondrial OXPHOS complex subunits were affected by palmitic acid, these complexes were resolved by second-dimension SDS-PAGE and subunits were detected using specific antibodies. Employing this procedure, the most striking finding was a fall in the amount of all studied complex subunits in cells treated with palmitic acid (Fig. 1E). No significant differences were observed whether subunits were encoded by genomic or mitochondrial DNA.

### Palmitic acid decreased gene expression of mitochondrial DNA (mtDNA)-encoded OXPHOS subunits

**Fig. 2.**
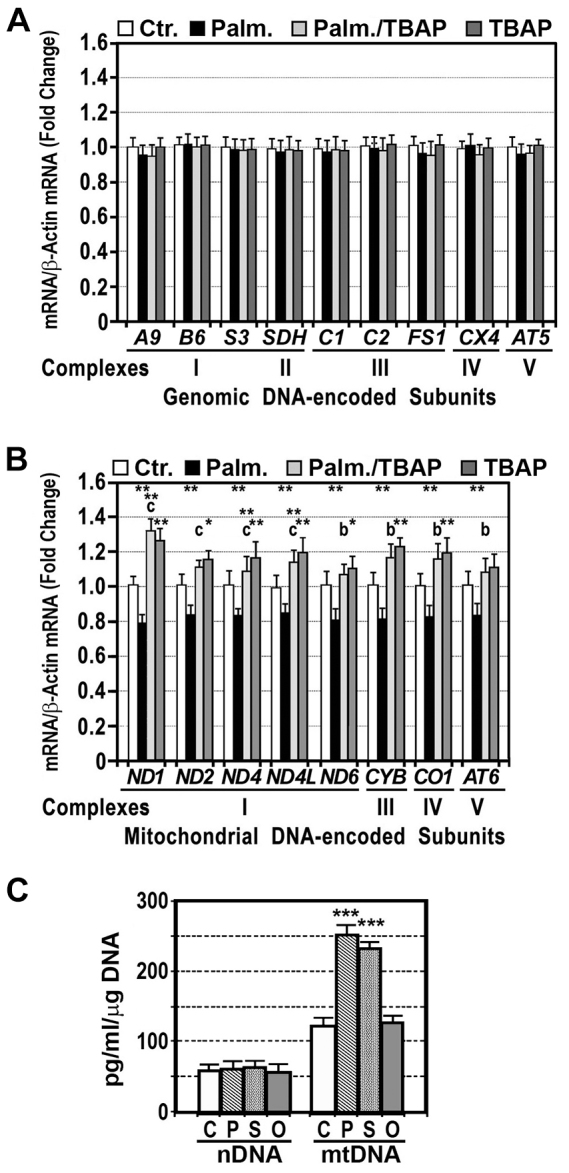
**Palmitic acid decreased gene expression of OXPHOS subunits.** (A,B) Gene expression of representative subunits encoded by genomic- and mitochondrial-DNA was measured in HepG2 cells treated with 200 μM palmitic acid (Palm.) for 24 hours in the absence or presence of 4 mM MnTBAP (TBAP). mRNA of the subunits was analyzed by RT-PCR following the procedure described in the Materials and Methods. The subunit mRNA:β-actin mRNA ratio was calculated. *P<0.05; **P<0.01 versus control untreated cells (Ctr.); b, *P*<0.01; c, *P*<0.001 versus cells treated with palmitic acid. *A9*, *NDUFA9*; *B6*, *NDUFB6*; *S3*, *NDUFS3*; *SDH*, *SDHA*; *C1*, *UQCRC1*; *C2*, *UQCRC2*; *FS1*, *UQCRFS1*; *AT5*, *ATP5A1*; *ND1*, *MTND1*; *ND2*, *MTND2*; *ND4*, *MTND4*; *ND4L*, *MTND4L*; *ND6*, *MTND6*; *CYB*, *MTCYB*; *CO1*, *MTCO1*; *AT6*, *MTATP6*. (C) HepG2 cells were cultured in the absence (C) or presence of 200 μM palmitic (P), stearic (S) or oleic (O) acids for 24 hours. 8-hydroxy-2′-deoxyguanosine (8-OHdG) content was measured in nDNA and mtDNA of the same cells. ***P<0.001 as compared with untreated cells.

Because a decrease in these subunits might be due to a diminished synthesis or to an accelerated degradation, we measured gene expression of some representative subunits of these complexes. We found that expression of genomic DNA (nDNA)-encoded subunits was normal in cells treated with 200 μM palmitic acid for 24 hours, and that preincubation with an antioxidant, such as MnTBAP, did not increase significantly the levels of these subunits (Fig. 2A). By contrast, gene expression of mtDNA-encoded subunits declined significantly in HepG2 cells treated with this fatty acid (Fig. 2B). Pretreatment of cells with MnTBAP increased gene expression of these subunits over the levels in control cells (Fig. 2B). Measurement of 8-hydroxy-2′-deoxyguanosine (8-OHdG) content in nDNA was identical in cells treated with fatty acids than in untreated cells (Fig. 2C). By contrast, this marker for oxidative DNA damage was significantly increased in mtDNA from cells treated with palmitic or stearic acids but not in those treated with the monounsaturated oleic acid.

### Palmitic acid accelerated degradation of OXPHOS complexes

**Fig. 3.**
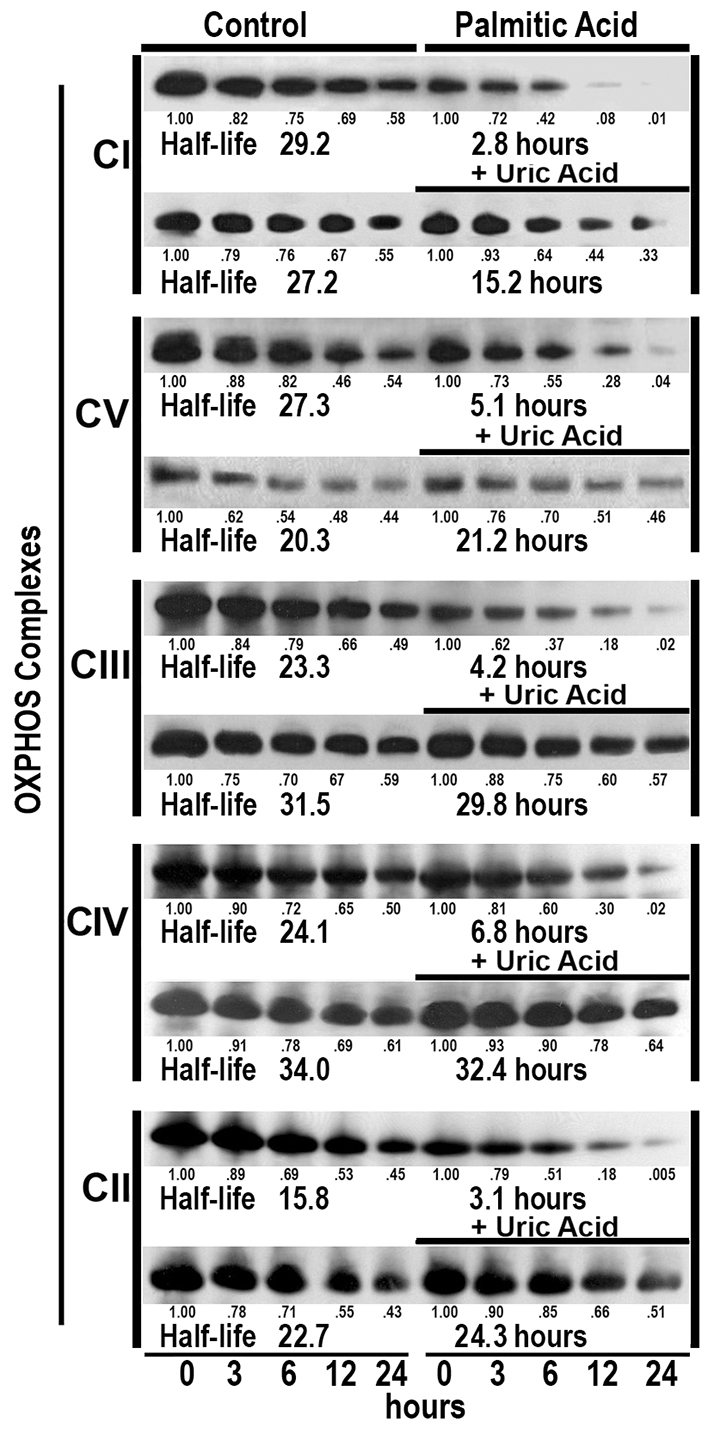
**Palmitic acid induced OXPHOS-complex degradation.** Confluent HepG2 cells were exposed to control medium or to 200 μM palmitic acid for 24 hours prior to inhibition of gene transcription with 5 μM actinomycin D. At 3, 6, 12 and 24 hours after the addition of actinomycin D, fully-assembled OXPHOS complexes were analyzed by BN-PAGE. The same experiments were repeated in the absence or presence of 200 μM palmitic acid or 1 mM uric acid. These experiments were repeated twice with similar results. Half-life values are in hours.

**Fig. 4.**
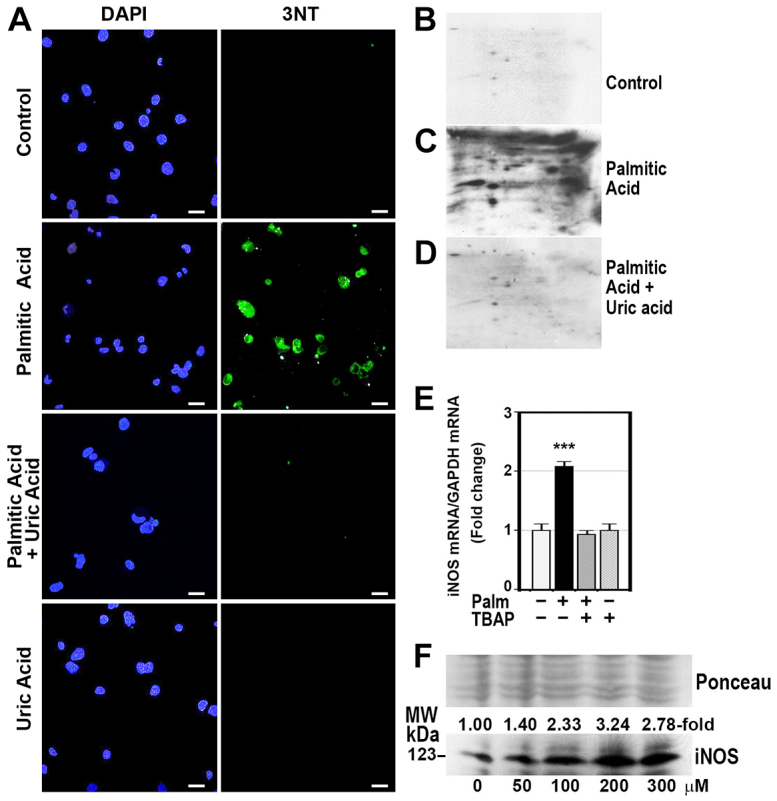
**Palmitic acid induced 3-tyrosine nitration of OXPHOS subunits.** (A) Confluent HepG2 cells were exposed to control medium or to 200 μM palmitic acid in the absence or presence of 1 mM uric acid for 24 hours. Cells were then fixed for 1 hour in 1% paraformaldehyde and permeabilized with Triton X before labelling with anti-3-nitrotyrosine (3NT)-specific antibody and goat anti-rabbit IgG FITC-conjugated secondary antibody. Nuclear counterstain of these cells was done with Fluoroshield (Sigma-Aldrich, Alcobendas, Spain) with DAPI. Scale bars: 30 μm. (B-D) Membranes containing mitochondrial proteins from control untreated HepG2 cells (B) or from cells treated with 200 μM palmitic acid (C) or with palmitic and 1 mM uric acid (D) were probed with specific antibody against 3NT. (E) Gene expression of *iNOS* was measured in HepG2 cells treated with 200 μM palmitic acid (Palm) for 24 hours in the absence or presence of 4 mM MnTBAP (TBAP). mRNA of iNOS was analyzed by RT-PCR following the procedure described in the Materials and Methods. The iNOS mRNA:GAPDH mRNA ratio was calculated. ***P<0.001 versus control untreated cells. (F) Western blots showing the effect of increasing doses of palmitic acid on iNOS protein expression.

In order to know whether saturated fatty acids caused degradation of complex proteins, confluent HepG2 cells were cultured in the absence or presence of 200 μM palmitic acid for 24 hours. After this time, gene transcription was inhibited by adding 5 μM actinomycin D. At 3, 6, 12 and 24 hours after addition of actinomycin D, fully-assembled OXPHOS complexes were analyzed by BN-PAGE. As Fig. 3 shows, palmitic acid decreased the half-life of OXPHOS complexes to about 18.8±6.6% of controls. This effect was associated with an increased amount of 3-tyrosine-nitrated proteins (Fig. 4A,C). Treatment of cells with 1 mM uric acid, a scavenger of peroxynitrite, prevented both the 3-tyrosine nitration of mitochondrial proteins (Fig. 4D) and the shortening of OXPHOS-complex half-life caused by palmitic acid (Fig. 3). Moreover, palmitic acid increased *iNOS* gene and protein expression (Fig. 4E,F).

### Saturated fatty acids induce oxidative stress

**Fig. 5.**
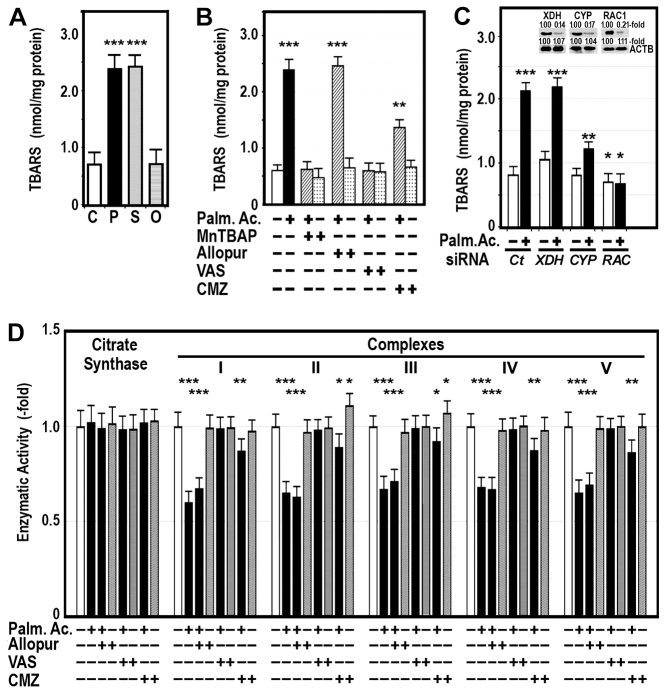
**Effects of fatty acid on TBARS contents in HepG2 cells.** (A) Cells were either untreated (C), or were pretreated with 200 μM palmitic acid (P), 200 μM stearic acid (S) or 200 μM oleic acid (O) for 24 hours. Cellular content of TBARS was measured as described in the Materials and Methods. (B) Cellular TBARS were measured in cells treated with 200 μM palmitic acid (Palm. Ac.) in the presence (+) or absence (−) of 4 mM MnTBAP, 0.3 mM allopurinol (Allopur), 10 μM VAS2870 (VAS) or 40 μM chlormethiazole (CMZ). (C) NADPH oxidase (*RAC1*), *CYP2E1* (*CYP*) or xanthine oxidase (*XDH*) were silenced with appropriate small interfering RNAs (siRNAs) in HepG2 cells in the presence (+) or absence (−) of 200 μM palmitic acid. TBARS content was measured in these cells. Western blots showing expression of XDH, CYP, RAC1 and β-actin (ACTB) after knocking down *XDH*, *CYP* or *RAC1*, respectively. Ct, control. (D) Enzyme activity of the OXPHOS complexes and citrate synthase was measured in HepG2 cells treated for 24 hours with 200 μM palmitic acid in the absence or presence of 0.3 mM allopurinol, 10 μM VAS2870 (VAS) or 40 μM chlormethiazole (CMZ). This activity was normalized to the level of the specific activity of citrate synthase, and expressed as fold difference of the activity in untreated cells. Values are expressed as mean ± s.d. *P<0.05; **P<0.01; ***P<0.001 compared with untreated cells.

Because nitro-oxidative stress seemed to be involved in the effects of saturated fatty acids on OXPHOS complexes, we wanted to know whether fatty acids are able to induce oxidative stress. As Fig. 5A shows, treatment of HepG2 cells with 200 μM palmitic or stearic acids for 24 hours led to a marked increase in the cellular levels of TBARS (thiobarbituric-acid-reacting substances), an index of oxidative stress. By contrast, treatment of cells with 200 μM oleic acid did not modify these levels.

In order to identify the oxidative system responsible for this stress, we treated HepG2 cells with 200 μM palmitic acid in the presence and absence of 4 mM MnTBAP, a mimic of superoxide dismutase, 0.3 mM allopurinol, 10 μM VAS2870 {1,3-benzoxazol-2-yl-3-benzyl-3H-[1,2,3]triazolo[4,5-d]pyrimidin-7-yl sulfide, 7-(1,3-benzoxazol-2-yl-sulfanyl)-3-benzyl-3H-[1,2,3]triazolo[4,5-d]pyrimidine, 7-(2-benzoxazolylthio)-3-(phenylmethyl)-3H-1,2,3-triazolo[4,5-d]pyrimidine} or 40 μM chlormethiazole, inhibitors of XDH, NADPHox and CYP2E1, respectively. As shown in Fig. 5B, the oxidative effect caused by palmitic acid was totally avoided by inhibiting NADPHox and partially by blocking CYP2E1. As expected, this effect was also prevented by treating cells with MnTBAP. By contrast, inhibiting XDH did not avoid the effects of palmitic acid on TBARS concentration. In order to ensure these results, we silenced *XDH*, *CYP2E1* or *NADPHox* with appropriate interfering RNAs (siRNA). These cells were then treated with palmitic acid. As Fig. 5C shows, the effects of palmitic acid persisted in cells with silenced *XDH*, but not in cells with silenced *RAC1*, a component of the NADPHox complex. Silencing *CYP2E1* reduced but did not abolish entirely the effect of palmitic acid on cellular TBARS.

### The effects of palmitic acid on OXPHOS activity were totally prevented by inhibiting or silencing NADPHox

Because NADPHox seemed to be involved in causing the oxidative stress induced by palmitic acid, we wanted to know whether NADPHox also mediated the inhibitory effects of palmitic acid on OXPHOS complexes. Thus, we treated HepG2 cells with 200 μM palmitic acid for 24 hours in the absence or presence of 0.3 mM allopurinol, 10 μM VAS2870 or 40 μM chlormethiazole. As Fig. 5D shows, the palmitic-acid-induced inhibition of OXPHOS complex activity was totally blocked by inhibiting NADPHox with VAS2870. In cells incubated with chlormethiazole, the effects of palmitic acid were partially reverted, whereas allopurinol pretreatment did not modify activity of these complexes.

**Fig. 6.**
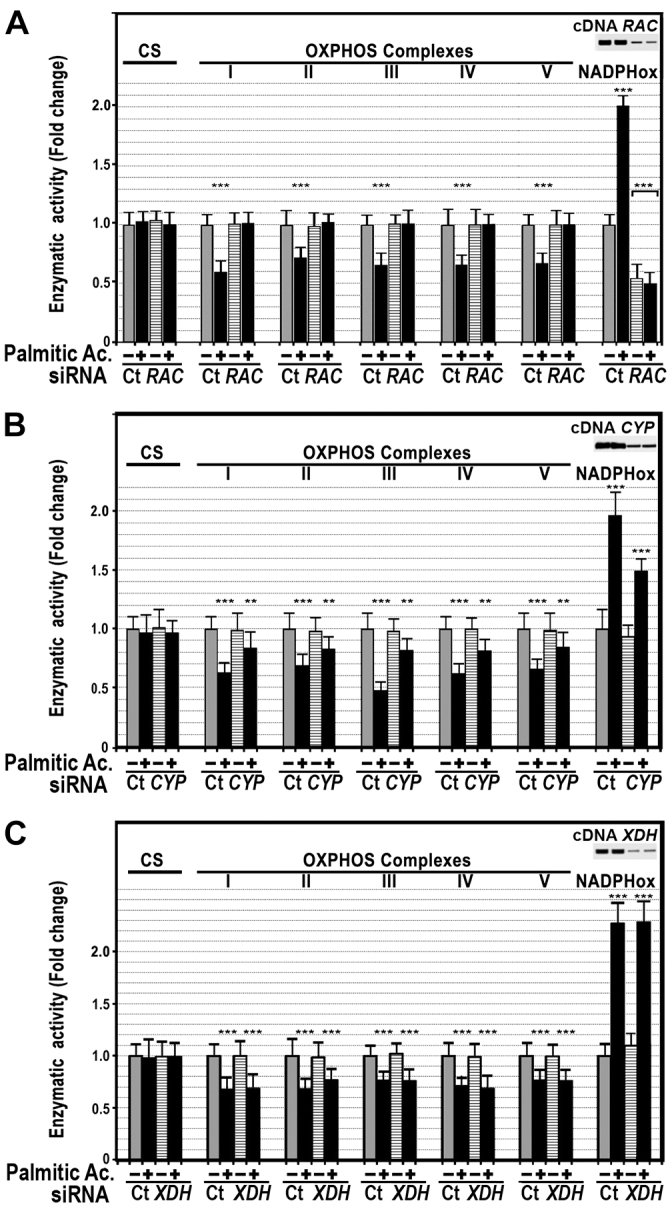
**The inhibitory effect of palmitic acid on OXPHOS activity was totally abrogated in the absence of NADPHox activity.** In HepG2 cells, *RAC1* (*RAC*) (A), *CYP2E1* (*CYP*) (B) or *XDH* (C) gene expression was silenced using appropriate siRNAs and activity of OXPHOS and NADPHox was measured in the presence and absence of 200 μM palmitic acid. **P<0.01; ***P<0.001 versus untreated control cells (siRNA Ct). CS, citrate synthase. In the top right-hand corner of each panel are northern blots showing cDNA levels for *RAC1* (A), *CYP* (B) and *XDH* (C) after knocking down expression of these genes, respectively.

To confirm that NADPHox mediated the effects of palmitic acid on OXPHOS complexes, we measured OXPHOS activity in cells with silenced *RAC1*, a component of the NADPHox complex. As Fig. 6A shows, the inhibitory effect of palmitic acid on the OXPHOS activity was not observed in the absence of NADPHox activity. This effect of palmitic acid persisted, but was less pronounced, in cells with silenced *CYP2E1*. However, in these cells, treatment with palmitic acid also increased NADPHox activity although less markedly (Fig. 6B). Finally, this effect persisted in cells with silenced *XDH* (Fig. 6C).

### Saturated free fatty acids increased NADPHox activity and gene expression in cultured HepG2 cells

**Fig. 7.**
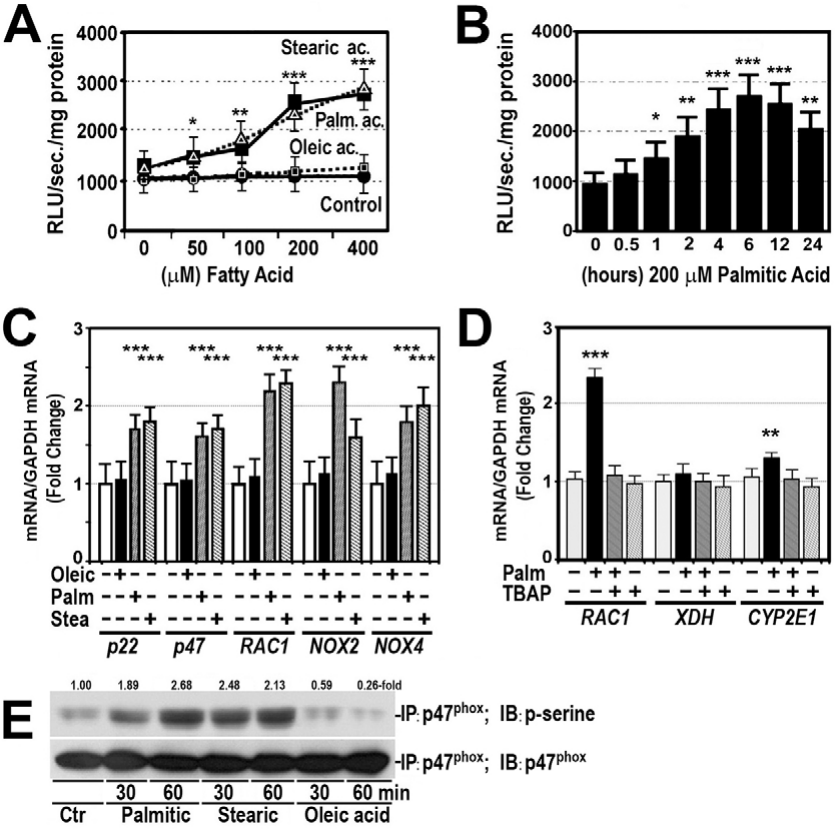
**Saturated fatty acids increase NADPHox activity and gene expression in cultured HepG2 cells.** (A) Cells were cultured in the presence of increasing doses of palmitic (triangles, broken line), stearic (squares, solid line) or oleic (squares, broken line) acids for 6 hours. Results for control cells are shown with black circles, solid line. NADPHox activity was measured as described in the Materials and Methods. RLU, relative light units. *P<0.05; **P<0.01; ***P<0.001. (B) NADPHox enzyme activity was measured in cultured HepG2 cells treated with 200 μM palmitic acid for 0.5 to 24 hours. *P<0.05; **P<0.01; ***P<0.001. (C) The effect of 200 μM palmitic, stearic or oleic acid on gene expression of *p22*, *p47^phox^* (*p47*), *RAC1*, *NOX2* and *NOX4* was analyzed by RT-PCR as described in the Materials and Methods. The mRNA:GAPDH mRNA ratio was measured in control cells and in cells treated with fatty acids for 24 hours. ***P<0.001 as compared with control cells. Results represent mean values ± s.d. of one representative experiment performed in quadruplicate. (D) The effect of 200 μM palmitic acid on *RAC1* (NADPHox), *XDH* and *CYP2E1* gene expression in the absence and presence of 4 mM MnTBAP. ***P<0.001, **P<0.01 as compared with control cells. Results represent mean values ± s.d. of one representative experiment performed in quadruplicate. (E) HepG2 cells were treated with 200 μM palmitic, stearic or oleic acids for 30 and 60 minutes. Cellular proteins were immunoprecipitated (IP) with anti-p47^phox^ (p47^phox^) and subsequently immunoblotted (IB) with either anti-phosphoserine (p-serine) or anti-p47^phox^ (p47^phox^) to evaluate equal loading.

Because NADPHox seemed to play a crucial role in the pathogenesis of oxidative stress caused by saturated fatty acids, we wanted to know whether these acids are able to activate this enzyme complex. Thus, we measured NADPHox activity in HepG2 cells treated with 200 μM palmitic, stearic or oleic acids. Although treatment of these cells with increasing doses of monounsaturated oleic acid did not modify significantly NADPHox activity, treatment with the same doses of palmitic or stearic acids led to a significant dose-dependent increase in this activity (Fig. 7A). Likewise, time-response curves demonstrate that palmitic acid increased NADPHox activity in a time-dependent fashion and that this effect was maximal by treating cells for 6 hours (Fig. 7B). Similarly, treatment of cultured HepG2 cells with 200 μM of either palmitic or stearic acids elevated significantly *p22^phox^*, *p47^phox^*, *RAC1*, *NOX2* and *NOX4* gene expression (Fig. 7C). By contrast, the same amount of oleic acid did not modify the expression of these genes significantly. Palmitic acid did not increase *XDH* gene expression but increased slightly *CYP2E1* gene expression (Fig. 7D). Moreover, saturated fatty acids not only upregulated gene expression of NADPHox components but also induced phosphorylation of p47^phox^, one component of the NADPHox. This effect was not observed with oleic acid (Fig. 7E).

## DISCUSSION

In the present study, we show for the first time that saturated fatty acids, but not the monounsaturated oleic acid, decreased markedly the activity of all OXPHOS complexes in HepG2 cells (Fig. 1A). As a result of this effect, ATP content (Fig. 1B) and ATP:ADP ratio were also significantly reduced in cells exposed to saturated fatty acid (Fig. 1C). Although we are not aware of other studies where the effects of fatty acids on the OXPHOS activity have been studied, some authors have reported that palmitic acid decreased cellular ATP content in muscle cells (Lambertucci et al., 2012; Hirabara et al., 2010). Our findings allow us to suggest that fatty acids, concentrations of which are increased in plasma and liver of individuals with NASH (Allard et al., 2008) and obese mice (García-Ruiz et al., 2014), could be responsible for the low OXPHOS enzyme activity we found in these individuals and animals (García-Ruiz et al., 2006; García-Ruiz et al., 2010; Solís-Muñoz et al., 2011). These effects of saturated fatty acids were blocked by the use of an antioxidant (Fig. 1A), indicating that oxidative stress might be implicated in these effects.

The present study also provides an explanation for the low activity of OXPHOS enzyme complexes in cells exposed to saturated fatty acids, because complex subunits and the amount of fully assembled complexes were markedly reduced in mitochondria of treated cells (Fig. 1D,E). This low amount of complex subunits might be caused by a reduced synthesis of these subunits, by their increased degradation or by a combination of both defects.

Our study shows that palmitic acid significantly decreased gene expression of mtDNA-encoded polypeptides, whereas expression of nDNA-encoded subunits was not affected by this acid (Fig. 2A,B). Therefore, a reduced synthesis of OXPHOS subunits can explain, at least partially, the low amount of mtDNA-encoded polypeptide found in cells exposed to palmitic acid, but not the reduced amount in nDNA-encoded subunits. These differences between both groups of proteins might be attributable to the oxidative damage of the mtDNA, because accumulation of mtDNA lesions might decrease the synthesis of mtDNA-encoded OXPHOS polypeptides. In fact, our study shows that gene expression of mtDNA-encoded subunits increased markedly by pretreating cells with an analog of superoxide synthase. Moreover, determination of the levels of 8-OHdG, a marker for oxidative DNA damage (Kasai 1997), demonstrated that 8-OHdG was significantly increased in mtDNA, but not in nDNA, of palmitic-acid-treated cells (Fig. 2C). mtDNA is particularly prone to suffer oxidative damage (Yakes and Van Houten, 1997) because it is not covered by protective histones and other DNA-associated proteins, allowing direct exposure to ROS. Moreover, mitochondrial DNA repair systems seem to be less efficient than those for nuclear DNA (Druzhyna et al., 2008; Gao et al., 2004). Finally, mtDNA is located near to the damaged OXPHOS chain, another major source of ROS. Therefore, mitochondrially generated ROS might subsequently lead to more mtDNA mutations and trigger a vicious cycle in which mitochondrial dysfunction produces larger amounts of ROS which in turn can induce further oxidative damage to mitochondrial function.

Our study also shows for the first time that palmitic acid accelerates degradation of OXPHOS complexes, reducing half-lives of fully-assembled complexes by about sixfold (Fig. 3). This effect seems to be dependent on the nitrosative stress, because pretreating cells with uric acid, a natural scavenger of peroxynitrite anion (Whiteman et al., 2002), prevented degradation of these complexes (Fig. 3). Moreover, mitochondrial proteins were 3-tyrosine nitrated in palmitic-acid-treated cells (Fig. 4C), and this acid increased *iNOS* gene and protein expression (Fig. 4E,F). In a previous study, we showed that ‘*in vitro*’ peroxynitrite caused not only nitration of mitochondrial proteins but also increased their degradation, decreased OXPHOS enzyme activity, reduced the amount of fully assembled OXPHOS complexes and reduced the amount of individual complex subunits (García-Ruiz et al., 2010). Also, Murray et al. demonstrated that ‘*in vitro*’ incubation of mitochondrial proteins with peroxynitrite inhibited complex I activity (Murray et al., 2003). This accelerated degradation of mitochondrial proteins justifies not only the low amount of mtDNA-encoded subunits, but also the decrease in nDNA-encoded subunits whose synthesis was normal. Peroxynitrite is produced by the reaction of nitric oxide (NO) with superoxide anion (O_2_^−^). A number of studies have shown that NO and superoxide anion formation is increased in the liver of individuals with NASH (Laurent et al., 2004; Sanyal et al., 2001) and obese mice (García-Ruiz et al., 2006).

Our study clearly shows that saturated fatty acids, but not the monounsaturated oleic acid, induced oxidative stress (Fig. 5A) and that this stress was totally abrogated by inhibiting NADPHox with VAS2872, a specific inhibitor of this oxidase (Fig. 5B), or by silencing *RAC1*, a component of NADPHox (Fig. 5C). We also show that palmitic and stearic acids, concentrations of which are significantly increased in the liver of obese mice (Wang et al., 2011), are able to markedly elevate NADPHox activity in HepG2 cells (Fig. 7A,B). Therefore, the oxidative stress found in HepG2 cells exposed to saturated fatty acids might be mediated mainly by the activation of NADPHox. This oxidase is a multiprotein complex composed of membrane-bound components (p22*^phox^*, Nox family) and cytosolic components (p47^phox^, p67^phox^, p40^phox^, Rac1/2) (De Minicis et al., 2006). Following stimulation, the cytosolic proteins become phosphorylated and are transferred to the membrane, where they bind to the membrane-bound components, increasing NADPHox activity and reducing molecular oxygen to generate superoxide and hydrogen peroxide. In HepG2 cells, our study shows that the increase in NADPHox activity was due to an upregulation of NADPHox-component gene expression (Fig. 7C) and to an enhanced phosphorylation of p47^phox^ (Fig. 7E). The effects of palmitic acid on gene expression of other oxidative systems, such as CYP2E1 or XDH, were less marked or even absent (Fig. 7D). There is no information about the effect of fatty acids on NADPHox activity in HepG2 cells. Nevertheless, a number of authors have shown that palmitate generated ROS via NADPHox in several cell lines (Han et al., 2012; Lambertucci et al., 2008).

NADPHox-dependent oxidative stress seems to be responsible for the depression in OXPHOS activity caused by palmitic acid, given that this effect did not occur in the presence of VAS2872 (Fig. 5D) or in cells with silenced NADPHox (*RAC1*) (Fig. 6). We have very little information about the effects of NADPHox on OXPHOS function. Nox4, one member of the Nox family located in the mitochondrial inner membrane, has been shown to inhibit activity of complex I of the OXPHOS and to decrease the concentration of complex I subunits (Kozieł et al., 2013). Gene expression of this factor is upregulated not only by transforming growth factor-β (Carmona-Cuenca et al., 2006), whose concentration is increased in the liver tissue of obese mice (García-Ruiz et al., 2014), but also by palmitic acid in HepG2 cells (Fig. 7C).

Other oxidative systems that could also contribute to the oxidative stress caused by saturated fatty acids are XDH and CYP2E1. The latter cytochrome is induced in humans and animals with NASH (Weltman et al., 1998; Lieber et al., 2004). Our study shows that inhibition or silencing CYP2E1 reduced oxidative stress (Fig. 5B,C) and prevented partially the effect of palmitic acid on OXPHOS complexes (Fig. 5D; Fig. 6), indicating that this cytochrome is also involved in the pathogenesis of oxidative stress caused by this fatty acid. However, these effects might be due to the cross-talk existing between CYP2E1 and NADPHox (Ekström and Ingelman-Sundberg, 1989). In fact, our study shows that palmitic-acid-induced NADPHox activity was decreased in cells with silenced CYP2E1 (Fig. 6). Other authors have found that inhibition of CYP2E1 with anti-CYP2E1 IgG antibody resulted in the complete inhibition of NADPHox-dependent generation of TBARS (Ekström and Ingelman-Sundberg, 1989). Likewise, induction of CYP2E1 resulted in an increased activity of NADPHox and oxidative stress, all of which could be prevented with chlormethiazole (Seitz and Mueller, 2012). Therefore, the improvement of OXPHOS function after inhibition of CYP2E1 might be attributable to the effects of CYP2E1 on the NADPHox system. In HepG2 cells, the role played by XDH in the fatty-acid-induced oxidative stress and inhibition of OXPHOS activity seems to be minor, because inhibition or silencing of this oxidase did not prevent the effects of palmitic acid on oxidative stress or OXPHOS-complex activity.

In conclusion, our study demonstrates that saturated fatty acids decrease OXPHOS enzyme activity by reducing the amount of OXPHOS complexes and their subunits. These effects are mediated by the nitro-oxidative stress caused by these acids, which results in reduced gene expression of mtDNA-encoded subunits, and in accelerated degradation of OXPHOS complexes. NADPHox and, to a lesser extent, CYP2E1 mediate these effects of fatty acids. Antioxidants and antiperoxinitrites prevent all these effects of fatty acids and might be useful in the treatment and prevention of NASH in humans.

## MATERIALS AND METHODS

### Cell culture

The HepG2 cell line obtained from American Type Culture Collection (Manassas, VA) was grown at 37°C in an atmosphere of 5% CO_2_, 95% air in cell culture flask using 10 ml of Dulbecco’s Modified Eagle’s Medium (Lonza Iberica SA, Barcelona, Spain) containing 10% fetal calf serum, 1% L-glutamine, 1% penicillin, 1% streptomycin, 1% fungizone. Cells were plated at a density of 5×10^6^/80-cm^2^ flask. The effect of fatty acid was examined by addition of these agents to the cell cultured in medium with 2% fetal calf serum. Palmitic and stearic fatty acids were dissolved as described by Joshi-Barve et al. (Joshi-Barve et al., 2007). Oleic acid was prepared according to the manufacturer’s protocol (Sigma-Aldrich, Alcobendas, Spain).

Nitration of cellular proteins by peroxinitrite [3-nitrotyrosine (3NT)] was assessed as described elsewhere (García-Ruiz et al., 2006).

### OXPHOS activity assays

HepG2 cells (approximately 5×10^6^ cells) were collected by trypsinization, washed twice with phosphate-buffered saline (PBS), and resuspended in 2 ml of ice-cold solution containing 20 mM MOPS, 0.25 M sucrose and 200 μg of digitonin. After centrifugation at 5000 ***g*** for 3 minutes at 4°C, the pellet was resuspended in 0.5 ml of 10 mM K-phosphate buffer, pH 7.4, and frozen-thawed twice. These digitonin-permeabilized homogenates were used to measure the activities of OXPHOS enzymes and citrate synthase (CS) using a DU-650 spectrophotometer (Beckman Instruments, Palo Alto, CA). Incubation temperatures were 30°C for complexes I, II, III, V and CS, and 38°C for complex IV. Enzyme activities were performed in supernatants as described elsewhere (Pérez-Carreras et al., 2003), expressed as nanomoles of substrate used per minute per milligram of protein and, to correct for the hepatic content of mitochondria, referred to as a percentage of the specific activity of CS. Enzyme assays were performed in triplicate.

### Quantitative real-time polymerase chain reaction

Total RNA was extracted from cultured HepG2 cells using the TRI-Reagent (Sigma-Aldrich, Steinheim, Germany) according to the manufacturer’s instructions. RNA was treated with DNase I to remove DNA contamination (Sigma-Aldrich, Steinheim, Germany). cDNA was generated from 1 μg sample RNA using First Strand cDNA Synthesis Kit for RT-PCR (Roche Applied Science, Indianapolis, IN) at 25°C, 5 min; 42°C, 60 min; 95°C, 5 min, and 4°C, 5 min. Quantitative real-time PCR was performed on a Light Cycler 1.0 (Roche Applied Science) in 20 μl with 50 ng cDNA, 0.5 μM primers and 2 μl FastStart DNA Master SYBR Green I (Roche Applied Science, Mannheim, Germany). Data from the real-time, quantitative PCR were analyzed following the 2-ΔΔCT method as described by Livak and Schmittgen (Livak and Schmittgen, 2001). The sequences of primers used in these experiments are shown in supplementary material Table S1. Expression of protein genes was normalized to that corresponding β-actin or *GAPDH* mRNA. The amplification conditions were 45 cycles of denaturation at 95°C for 10 s, annealing at 59°C for 5 s and extension at 72°C for 20 s (Bustin, 2000). The correct size and purity of the amplified products was verified by agarose gel electrophoresis.

### RNA interference

*XDH*-, *CYP2E1*- and *Rac1*-specific siRNA and non-specific control RNA used as a negative control were purchased from Santa Cruz Biotechnology, Inc. (Santa Cruz, CA). For the transfection experiments, we followed the procedure described elsewhere (Díaz-Sanjuán et al., 2009).

### Western blot

Mitochondria were isolated from cultured cells by differential centrifugation as described by Turko et al. (Turko et al., 2001). Proteins were separated and transferred to an Immobilon membrane (Millipore, Bedford, MA) as previously described (Solís-Herruzo et al., 1999). After electrotransfer, the filters were incubated with appropriate polyclonal antibody against 3-nitrotyrosine (Upstate Biotechnology, Lake Placid. NY), inducible nitric oxide synthase (iNOS), p47^phox^, TOM20, XDH, CYP2E1, Rac1, VDAC1 (Santa Cruz Biotechnology, Santa Cruz, CA) and phosphorylated serine (Sigma-Aldrich, Alcobendas, Spain). Signals were detected using the ECL Western Blotting Detection Reagent (Amersham Ibérica, Madrid, Spain).

### Immunoprecipitation

The immunoprecipitation assays were performed as previously described (Lang et al., 2000). Proteins were precipitated with appropriate polyclonal antibodies (anti-p47^phox^). Immune complexes were recognized using specific antibodies (p47^phox^, anti-phosphoserine). Signals were detected using the ECL detection kit.

### Assessment of fully-assembled OXPHOS complexes

Mitochondria were isolated from HepG2 cells according to the procedure described by Nijtmans et al. (Nijtmans et al., 2002). Mitochondrial complexes were separated on a 3–12% acrylamide blue native– polyacrylamide gel (BN-PAGE) as described elsewhere (García-Ruiz et al., 2010). Western blotting of these proteins was performed using primary antibodies against complex I subunit NDUFA9, complex II subunit SDHA, complex III subunit UQCRC2, complex IV subunit MTCO1, complex V subunit ATP5A1 (Molecular Probes Inc., Eugene, OR) and TOM complex subunit TOM20 on blocking buffer for 2 hours. After washing, blots were incubated for 1 hour with peroxidase-conjugated anti-mouse antibody as a secondary antibody, prepared at a 1:5000 dilution (Molecular Probes Inc., Eugene, OR). Immunoreactive material was visualized by chemiluminescence (ECL, Western Blotting Detection, GE Healthcare, Madrid, Spain) according to the manufacturer’s instructions. Blots were finally exposed to Hyperfilm MP (Amersham, GE Healthcare, Madrid, Spain). ECL signals were quantified using the ImageJ image analysis software (Rasband, 2007).

### Second-dimension electrophoresis for assessing complex subunits

For second-dimension BN/SDS-PAGE, a lane containing mitochondrial complexes was excised from the one-dimension gel, as previously described (García-Ruiz et al., 2010). Western blotting was performed using primary antibodies against: VDAC1 (Santa Cruz Biotechnology, Santa Cruz, CA); complex I subunits NDUFV1, NDUFV2, NDUFA6, NDUFB6, NDUFA9, NDUFS3, NDUFB8, MTND1, MTND4L and MTND6; complex II subunit SDHA; complex III subunits UQCRC1 (Core1), UQCRC2 (Core2), UQCRFS and MTCYB; complex IV subunits COX4 and MTCO1; and complex V subunits ATP5A1 and MTATP8 (Molecular Probes Inc., Eugene, OR) on blocking buffer for 2 hours. After washing, blots were treated as indicated above.

### Measurement of total ATP content and ATP:ADP ratio in mouse liver

Cells were homogenized in perchloric acid and centrifuged at 15,000 ***g*** for 2 minutes. Supernatants were collected and 30 μl was added to a 96-well plate and then brought up to 50 μl with ATP assay buffer. ATP reaction mix and ATP measurement was performed using the ATP Colorimetric/Fluorometric Assay Kit (BioVision Research Products, Milpitas, CA) according to the manufacturer’s protocol. The ATP:ADP ratio was measured by luminometry using the commercial assay kit ApoSENSOR™ ADP/ATP Ratio Assay Kit (BioVision Research Products, Mountain View, CA).

### Measurement of 8-OHdG in nuclear and mitochondrial DNA

Nuclear DNA (nDNA) and mitochondrial DNA (mtDNA) were isolated from HepG2 cells using genomic and mitochondrial DNA isolation kits according to the manufacturer’s protocol (BioVision Research Products, Mountain View, CA). Oxidative damage to nDNA and mtDNA was determined by measuring 8-OHdG using a competitive enzyme immune assay following the manufacturer’s indications (8-Hydroxy-2-deoxyguanosine EIA Kit, Cayman Chemical Co., Ann Arbor, MI).

### Lipid peroxidation

Lipid peroxidation was determined by measuring TBARS in cells as described by Ohkawa et al. (Ohkawa et al., 1979).

### NADPH oxidase activity

NADPHox activity was measured following the procedure described by Jalil et al. (Jalil et al., 2005).

### Statistical analysis

These analyses were carried out using the SPSS Statistical Software for Windows, version 9 (SPSS Inc., Chicago, IL). The unpaired *t*-test was used to assess the significance of differences between means. All results were expressed as mean ± s.d. *P*-values <0.05 were considered significant.

## Supplementary Material

Supplementary Material
