# Supplementary material for: In vitro treatment of HepG2 cells with saturated fatty acids reproduces mitochondrial dysfunction found in nonalcoholic steatohepatitis
Source: Dis Model Mech. 2014 Dec 24;8(2):183–91. doi: 10.1242/dmm.018234 (PMC4314783; doi:10.1242/dmm.018234)
Supplement: Supplementary Material [file supp_8_2_183__index.html]

In vitro treatment of HepG2 cells with saturated fatty acids reproduces mitochondrial dysfunction found in nonalcoholic steatohepatitis — Supplementary Material 

# *In vitro* treatment of HepG2 cells with saturated fatty acids reproduces mitochondrial dysfunction found in nonalcoholic steatohepatitis

## DMM018234 Supplementary Material

**Files in this Data Supplement:**

- **Supplementary Material**
