# Supplementary material for: In vitro treatment of HepG2 cells with saturated fatty acids reproduces mitochondrial dysfunction found in nonalcoholic steatohepatitis
Source: Dis Model Mech. 2014 Dec 24;8(2):183–91. doi: 10.1242/dmm.018234 (PMC4314783; doi:10.1242/dmm.018234)
Supplement: Supplementary Material [file supp_8.2.183_DMM018234.pdf]

Supplemental table S1. Primers used in quantitative real-time polymerase chain reaction

Primer name	Direction	Sequence
Human *NDUFA9*	Sense	5′-GAT TGT GGC CAC TGT GTT TGG-3′
	Antisense	5′-CTC CAG CTT CCT TGG ACA GT-3
Human *NDUFB6*	Sense	5′-CTG CAG CAG CTG CGA GA-3′
	Antisense	5′-GAA TAA TCC AGA CAG GTA CAA G-3′
Human *NDUFS3*	Sense	5′-GTC AGA CCA CGG AAT GAT GTG-3′
	Antisense	5′-CTC AAA ACG GTT TTG CCG AG-3′
Human *MTND1*	Sense	5′-CTT AGC TCT CAC CAT CGC TCT T-3′
	Antisense	5′-AGA TTG TTT GGG CTA CTG CTC G-3′
Human *MTND2*	Sense	5′-CTT AGC ATA CTC CTC AAT TAC CCA-3′
	Antisense	5′-TGG ATG GAA TTA AGG GTG TTA GTC-3′
Human *MTND4*	Sense	5′-CTC CAC TTA TGA CTC CCT AAA G-3′
	Antisense	5′-TGA TTG AAG AGT ATG CAA TGA GCG-3′
Human *MTND4L*	Sense	5′-GCA TTT ACC ATC TCA CTT CTA GG-3′
	Antisense	5′-GTT GGA GAT TGA GAC TAG TAG G-3′
Human *MTND6*	Sense	5′-ATC CTC CCG AAT CAA CCC TG-3
	Antisense	5′-GAT GGT TGT CTT TGG ATA TA-3′
Human β-*actin*	Sense	5′-GGA CGA CAT GGA GAA AAT CTG GCA-3′
	Antisense	5′-GTA GAT GGG CAC AGT GTG GGT G-3′
Human *GADPH*	Sense	5′-GCC AAA AGG GTC ATC ATC T-3′
	Antisense	5′-ATG GAT GAC CTT GGC CAG-3′
Human *UQCRC1*	Sense	5′-GTT AGC CTG CTG GAC AAC G-3′
	Antisense	5′-CTT GAT GTA GTA AGC TGT GTG C-3
Human *UQCRC2*	Sense	5′-CCA AAT GGC TTG GTG ATT GC-3′
	Antisense	5′-CAT TAG AAT ATC AAC ATC ACC CC-3
Human *UQCRFS*	Sense	5′-GCC TGT GTT GGA CCT GAA G-3′
	Antisense	5′-CTG GAA ACG AAC TGG GTG AC-3
Human *MTCYB*	Sense	5′-GAT CCT CCA AAT CAC CAC AGG AC-3′
	Antisense	5′-GGA GGA TAA TGC CGA TGT TTC AG-3′
Human *SDHA*	Sense	5′-GGC TTG CGA GCT GCA TTT GG-3′
	Antisense	5′-GTT CTG CTA AAC GGC ATG CCA-3′
Human *COX4*	Sense	5′-CAG GGT ATT TAG CCT AGT TGG-3′
	Antisense	5′-CTC CTT GAA CTT AAT GCG ATA C-3′
Human *MTCO1*	Sense	5′-GAG CTG GGC CAG CCA GGC AA-3′
	Antisense	5′-GGA AAC GCC ATA TCG GGG GCA-3′
Human *ATP5A1*	Sense	5′-GTA TTG GTG ATG GTA TTG CCC GC-3′
	Antisense	5′-AAC AGC TCC TCA CCA ACT GGA AC-3′
Human *MTATP6*	Sense	5′-GGC TTTC GCT CTA AGA TTA AAA ATG-3′
	Antisense	5′-GTA GAG GGA AGG TTA ATG TG-3′
Human *iNOS*	Sense	5′-TGT TTG AAC ACA TCT GCA GAC ACG-3′
	Antisense	5′-CAA GGT CAG GTG GGA TTT CGA AGA-3′
Human *CYP2E1*	Sense	5′-GCC TAC GAC TGT TGT GAA CAC TG-3′
	Antisense	5′-CAA GAG GCT GCT GCG TCT GCA-3′
Human xanthine oxidase (*XDH*)	Sense	5′-CTG AGG CTG AAA GAC ACT CCT C-3′
	Antisense	5′-GAA GCT TAG CAA CAG CAT CCA CC-3′
Human *NOX4*	Sense	5′-CTT CTC TTC ACA ACT GTT CCT GGC-3′
	Antisense	5′-GAG CTG GTT CGG TTA AGA CTG ATG-3′
Human *NOX2*	Sense	5′-GAG TTT CAA GAT GCG TGG AAA CT-3′
	Antisense	5′-AGA TCT GCA AAC CAC TCA AAG G-3′
Human *p47*^{phox}	Sense	5′-CCG AGA TCT ACG AGT TCC AT-3′
	Antisense	5′-GAG CTT GAG GTC ATC AGG G-3′
Human *p22*^{phox}	Sense	5′-TTT GTG TGC CTG CTG GAG T-3′
	Antisense	5′-AGT GGA TAG ATG CCG CTC G-3′
Human *RAC1*	Sense	5′-GTG AAT CTG GGC TTA TGG GAT-3′
	Antisense	5′-GTG ATG GGA GTC AGC TTC TT-3′
